# A resected case of recurrent ITPN in the remnant pancreas after pancreatoduodenectomy

**DOI:** 10.1186/s40792-019-0590-0

**Published:** 2019-02-19

**Authors:** Kenju Ko, Yasunori Nishida, Kotaro Sasahara, Hirofumi Kishimoto, Otagiri Noriaki, Katsunori Tauchi, Koji Azuhata, Kayoko Higuchi

**Affiliations:** 1 Department of Surgery, Aizawa Hospital, 2-5-1 Honjo, Matsumoto, Nagano 390-8510 Japan; 2 Department of Pathology, Aizawa Hospital, 2-5-1 Honjo, Matsumoto, Nagano 390-8510 Japan; 3 Department of Pathology, Okinawa Kyoudo Hospital, 4-10-55 Kohagura, Naha, Okinawa 900-8558 Japan

**Keywords:** Intraductal tubulopapillary neoplasm (ITPN), Recurrence, Remnant pancreas

## Abstract

**Background:**

Since intraductal tubulopapillary neoplasm (ITPN) is a rare disease, the clinical features of ITPN, especially the characteristics related to recurrence, have not been revealed. We performed a total remnant pancreatectomy for a patient whose ITPN recurred 16 months after pancreatoduodenectomy (PD). We report useful findings to clarify how ITPN reoccurs based on this experience and previously reported cases.

**Case presentation:**

A 61-year-old male patient was diagnosed with pancreatic cancer and underwent PD. However, a postoperative pathologic examination diagnosed ITPN with invasive cancer. After receiving adjuvant chemotherapy, he was hospitalized for pancreatitis 16 months after the operation. He was diagnosed as having recurrence near the pancreato-jejunal anastomosis based on detailed examinations and underwent a remnant total pancreatectomy. From the results of the histopathological examination, he was found to have a recurrence of ITPN as a polypoid mass without invasion distant from the surgical stump of the first operation. Furthermore, tumor cells floating in the main pancreatic duct distant from the main tumor were observed at three locations.

**Review of the literature:**

Including our case, five cases of recurrence in the remnant pancreas after surgery for ITPN have been reported. Recurrence in the main pancreatic duct was observed in four of these five cases. The primary tumor, which recurred in the remnant pancreas after surgery, was characterized as being relatively small and less invasive; however, Ki-67 labeling index was high. In immunohistochemical examination, the expression of MUC6, which is not one of characteristics of ITPN, tended to be positive.

**Conclusion:**

In this case, tumor cells were floating inside the pancreatic duct at several locations. From the results of this case and a review of previous reports, the cause of ITPN recurrence in this case seemed to be due to tumor cells leaving the tumor and implanting into the pancreatic duct.

## Background

According to the current classification of the World Health Organization (WHO) [1], intraductal tubulopapillary neoplasm (ITPN) is defined as a subtype of intraductal neoplasms of the pancreas. The clinical features of ITPN have not been revealed due to its rarity; it is composed of less than 1% of exocrine pancreas tumors. Therefore, the characteristics related to recurrence are also unknown as there are only a few reports. We experienced a remnant pancreatectomy for a patient whose ITPN recurred 16 months after pancreaticoduodenectomy (PD). We report on useful findings to clarify how ITPN reoccurs based on this case.

## Case presentation

A 61-year-old male patient without a previous medical history was diagnosed with pancreatic cancer in August 2015 and underwent PD. However, a postoperative pathologic examination yielded a diagnosis of ITPN with associated invasive carcinoma. The patient underwent routine examinations after receiving postoperative chemotherapy with S-1 for 6 months (60 mg, orally administered twice a day for 28 days followed by a 14-day rest period). During hospitalization due to acute pancreatitis in December 2016, ITPN recurrence was diagnosed by detailed examinations.

Although he used to smoke 20 cigarettes and drink 700 ml of beer a day, the patient quit smoking and drinking after the first surgery. There was no remarkable past history.

### First surgery for primary ITPN

Laboratory data were normal, except for amylase (298 UI/I; normal, 10-20 UI/l) and lipase (352 UI/I; normal, 10-20 UI/I). Regarding tumor markers, carbohydrate antigen 19-9 (CA19-9) was slightly increased at 37.3 U/ml, but carcinoembryonic antigen (CEA) and DUPAN-2 were within normal limits.

**Fig. 1:**
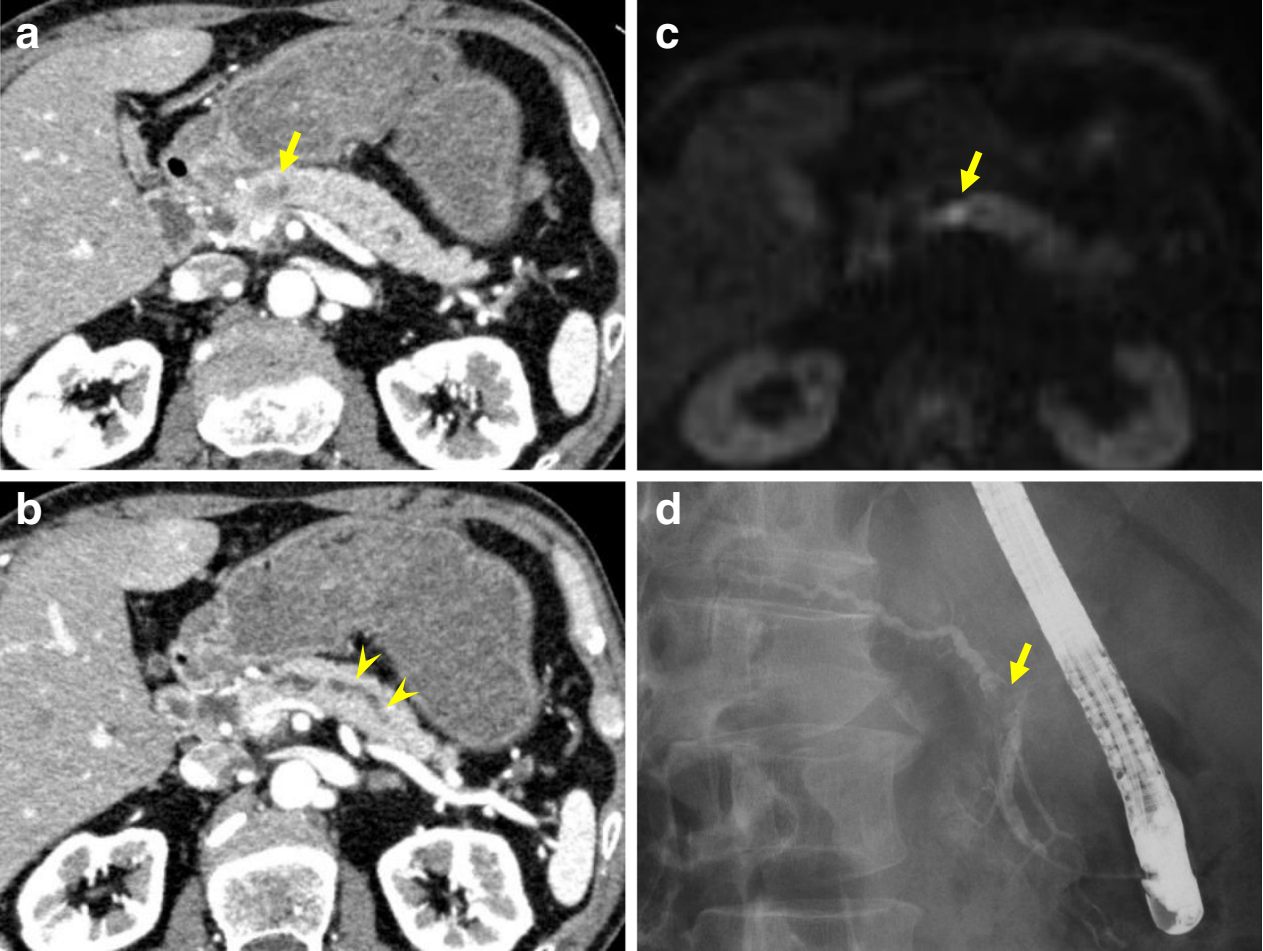
Preoperative image findings in the first surgery. **a**, **b** CT showed a low-contrast tumor approximately 1 cm (arrow) in size and dilatation of the upstream main pancreatic duct (arrowhead). **c** MRI revealed a tumor showing a high signal by DWI at the stenosis of the pancreatic duct. **d** ERCP showed an irregular defect in the main pancreatic duct

Preoperative computed tomography (CT) showed a tumor with a low-contrast effect approximately 1 cm in the head of the pancreas and dilatation of the upstream main pancreatic duct (Fig. 1a, b). At the stenosis of the pancreatic duct, there was a tumor that showed a low signal by fat suppression T1WI and a high signal by T2WI and diffusion-weighted imaging (DWI) (Fig. 1c).

Endoscopic retrograde cholangiopancreatography (ERCP) was performed before the operation. ERCP showed an irregular defect in the main pancreatic duct at the head of the pancreas (Fig. 1d). No image suggested mucus in the pancreatic duct. Brush cytology of the stenosis revealed only pancreatic duct epithelial cells with low atypia.

Given that pancreatic cancer was diagnosed based on these examinations, subtotal stomach-preserving pancreatoduodenectomy (SSPPD) was performed in August 2015.

**Fig. 2:**
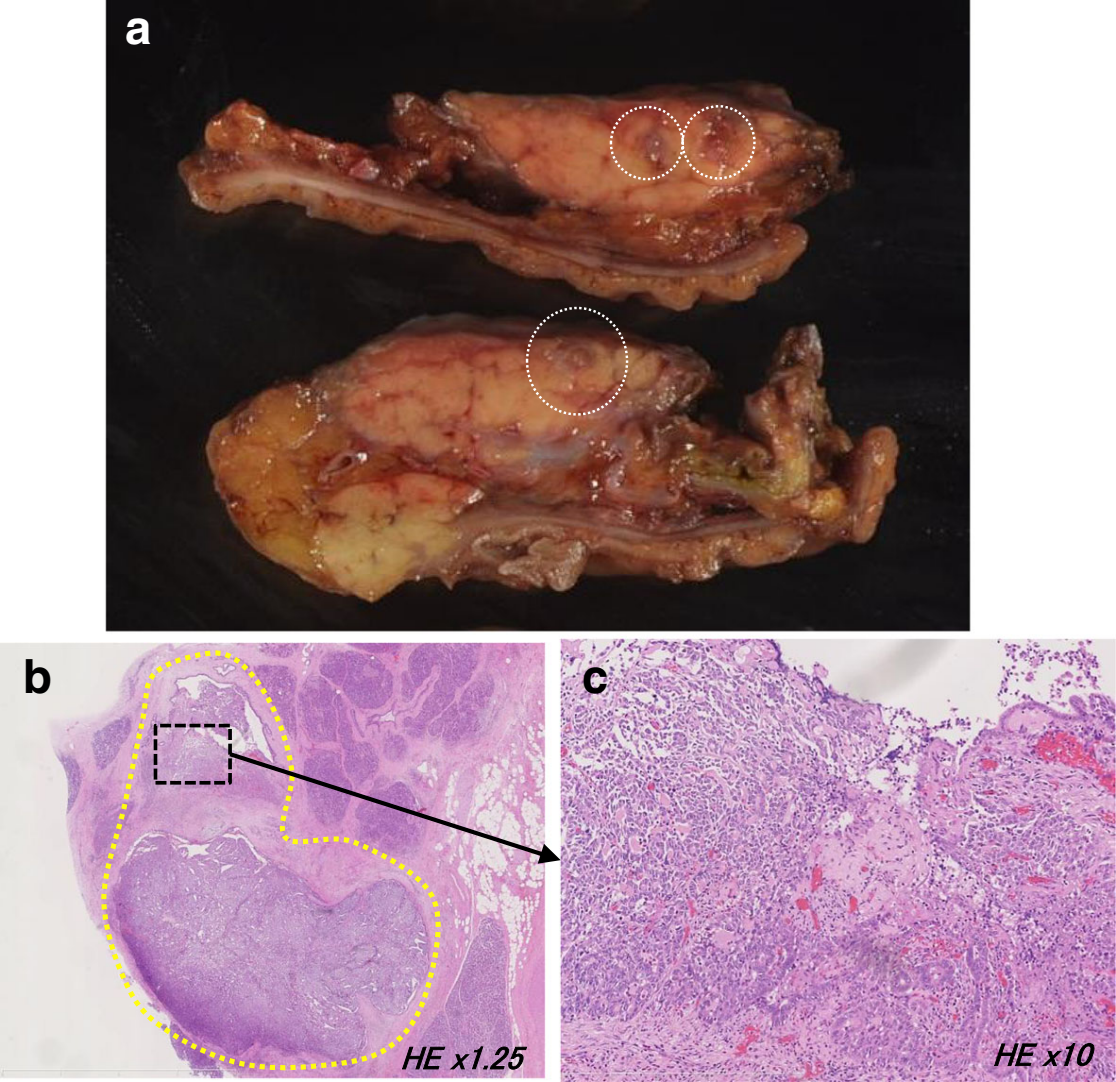
Macroscopic and histopathological findings of the resected specimen in the first surgery. **a** Macroscopic findings showed that the tumor filled the pancreatic duct. **b**, **c** There was a tumor growing with tubular or cribriform features in the vascular stroma at the main pancreatic duct. The tumor was accompanied by necrosis in some locations and invaded the stroma around the main pancreatic duct

The macroscopic findings of the resected specimen showed that the tumor filled the pancreatic duct (Fig. 2a). A tumor was growing with tubular or cribriform features in the vascular stroma at the main pancreatic duct. The tumor was accompanied by necrosis in some locations and invaded the stroma around the main pancreatic duct (Fig. 2b, c). Mucus production from the tumor was not observed. The results of immunohistochemical staining were as follows: cytokeratin7 (+), cytokeratin19 (+), MUC5AC (−), MUC2 (−), MUC6 (+), chromogranin A (−), synaptophysin (−), and P53 (+). The Ki-67 labeling index was 35.3%, resulting in a final diagnosis of ITPN with associated invasive carcinoma. A histopathological examination revealed no ITPN at the resection stump of the pancreas.

### Second surgery for recurrence

When recurrence was diagnosed, amylase and lipase levels were as high as 269 UI/I and 784 UI/I, respectively, but the other data were within normal limits. Each tumor marker, such as CEA, CA 19-9, and DUPAN-2, was within normal limits.

**Fig. 3:**
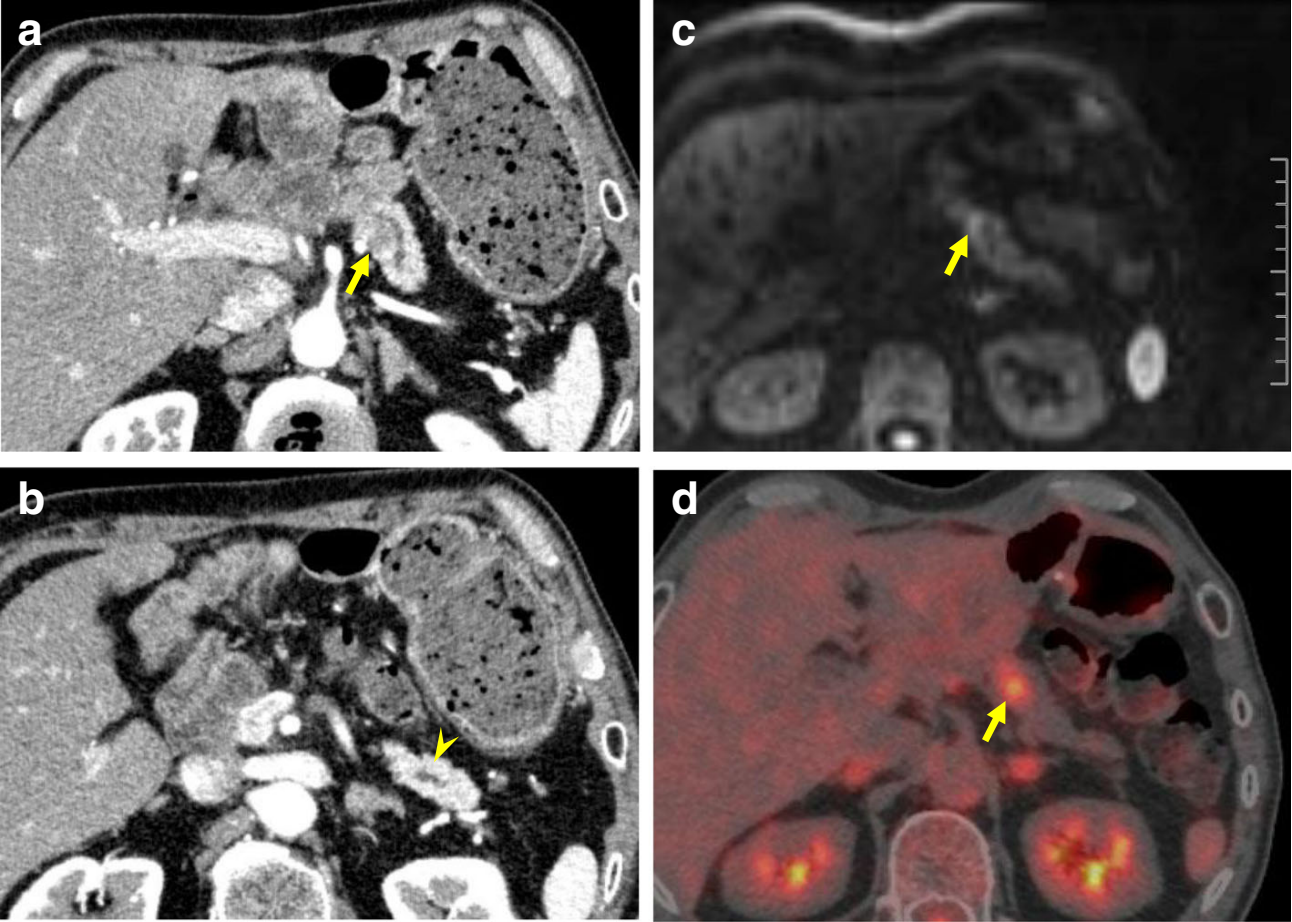
Preoperative image findings in the second surgery. **a** CT showed a low concentration region of 2 cm in size near the pancreato-jejunal anastomosis (arrow). **b** Dilatation of the upstream main pancreatic duct was observed (arrowhead). **c** MRI showed a tumor with a high signal by DWI at the stenosis of the pancreatic duct. **d** PET-CT revealed the accumulation of SUVmax 4.0 at the tumor

The CT showed a low concentration region of 2 cm in size near the pancreato-jejunal anastomosis, which was similar to the primary ITPN (Fig. 3a). Dilatation of the upstream main pancreatic duct was observed (Fig. 3b). MRI revealed a tumor showing a high signal by DWI at the stenosis of the pancreatic duct (Fig. 3c). Positron emission tomography/computed tomography (PET-CT) revealed an accumulation of SUV max 4.0 at the tumor, while any findings suggestive of other metastases were not observed (Fig. 3d).

As ITPN was not observed at the resection stump of the pancreas by pathological examination of the first surgery, the tumor was diagnosed as recurrence after complete resection rather than residual ITPN in the first operation. A total remnant pancreatectomy was performed in January 2017.

**Fig. 4:**
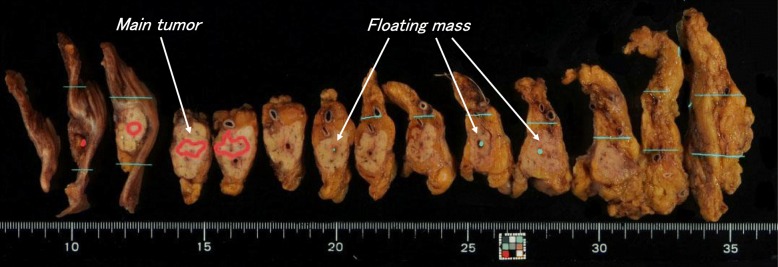
Resected specimens in the second surgery. Recurrence of ITPN was observed in the pancreatic duct at a distance of 2.5 cm from the pancreatic stump. Three tumor masses were observed in the main pancreatic duct at sites distant from the main tumor

**Fig. 5:**
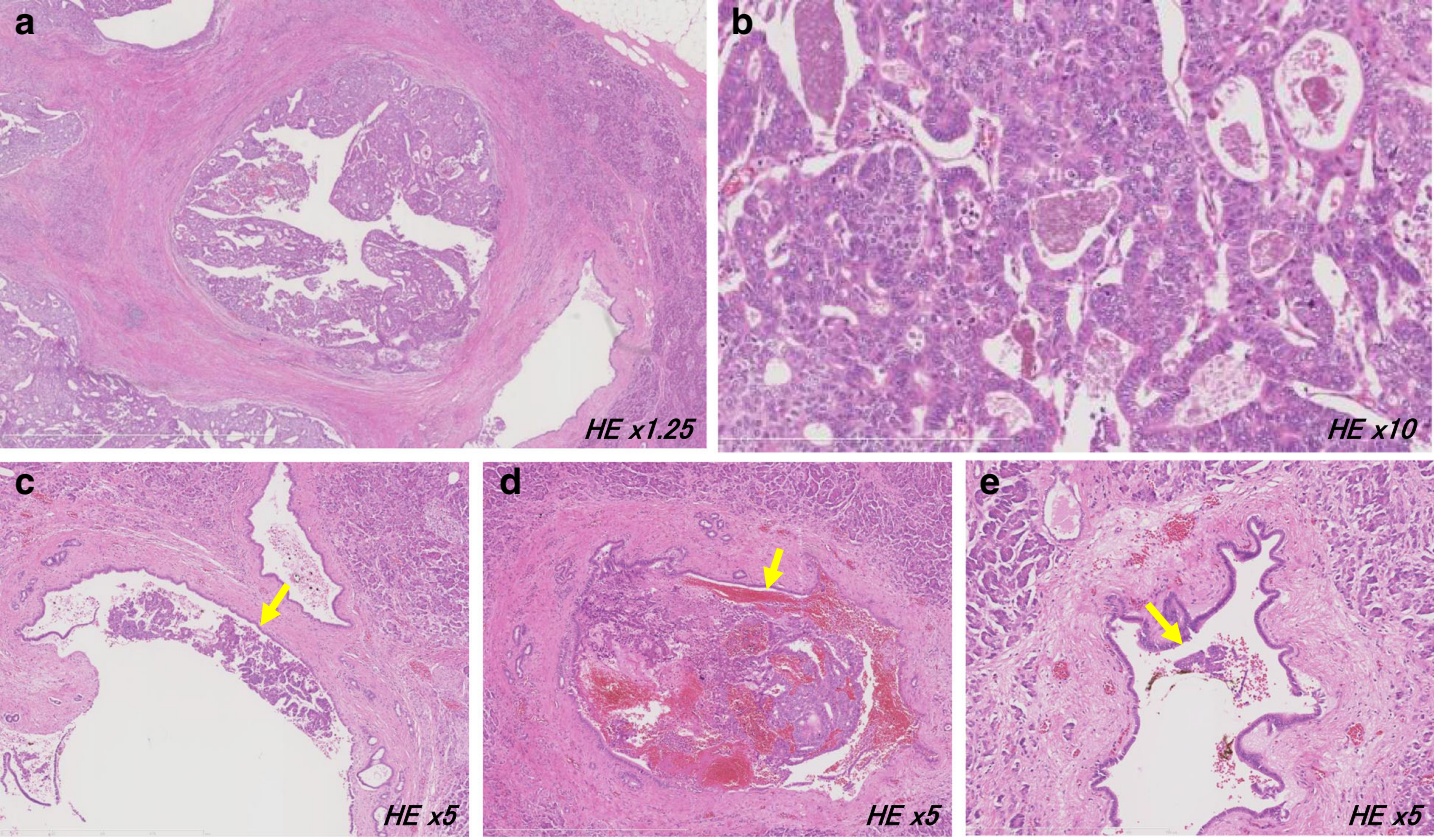
Histopathological findings of the resected specimens in the second surgery. **a**, **b** The main tumor infiltrated into the main duct and the surrounding branch of the pancreatic duct. **c**–**e** Each tumor mass floated in the main pancreatic duct (arrow). No obvious malignant finding was observed in the pancreatic duct epithelium at each region

In the histopathological investigation, ITPN polypoid recurrence in the pancreatic duct was observed at a distance of 2.5 cm from the pancreatic stump. Moreover, three tumor masses were observed in the main pancreatic duct at sites distant from the main tumor (Fig. 4). The main tumor did not show invasion but infiltrated into the main duct and the branches of the pancreatic duct (Fig. 5a, b).

Immunohistochemical staining results revealed cytokeratin7 (+), cytokeratin19 (+), MUC5AC (+/−), MUC2 (−), MUC6 (−), chromogranin A (+/−), synaptophysin (+/−), and p53 (+), which were similar to the primary ITPN. The expression of trypsin, which is indicative of acinar cell carcinoma, was negative. The Ki-67 labeling index was 45.1%, which was very similar to the 35.3% of the primary lesion. Three tumor masses were observed floating in the main pancreatic duct at sites distant from the tumor, while there were no obvious malignant findings in the pancreatic duct epithelium at each region (Fig. 5c–e).

Recurrence was not observed for 23 months after the second surgery.

## Discussion

ITPN was reported by Yamaguchi et al. [2] as a subclass of the pancreatic ductal tumor and classified separately from intraductal papillary mucinous neoplasm (IPMN) in the 2010 revision of the WHO classification [1]. Although the prognosis of ITPN is better than that of pancreatic cancer [1, 3], surgical resection is basic treatment because infiltration and metastasis often occur. Date et al. [4] reported that the prognosis after resection of ITPN was good because the 5-year survival rate was 80.7%. Furthermore, Date et al. compared cases with and without infiltration and concluded that there was no difference in the 5-year survival rate if the recurrent lesions were completely removed. These results clearly show a better prognosis of resected ITPN compared with invasive pancreatic cancer.

Although the prognosis of ITPN is much better than that of invasive pancreatic cancer, ITPN may reoccur after resection. Following the report by Yamaguchi et al. in 2009, nine cases of detailed descriptions on recurrence after resection have been published, including our case [2, 5–10]. Interestingly, recurrence in the remnant pancreas was observed in five of nine cases (Table 1). In reviewing these five cases, we noticed that each primary lesion was less invasive and small in size, although the Ki-67 labeling index was high in the pathological diagnosis of primary ITPN. ITPN with infiltration is not rare because the invasion was observed in 54 to 71% of ITPN cases in previous reports [4, 11, 12]. Less invasion of the primary ITPN may be one of the characteristics of recurrence in the remnant pancreas after surgery.

**Table 1:** Reported cases of recurrence in the remnant pancreas after surgery for ITPN

Case	Author	Year	Age	Gender	Primary ITPN					Recurrent ITPN					
					Location	Size (mm)	Surgery	Ki-67	Invasion	Time to recurrence (m)	Location	Size (mm)	Treatment	Survival after first surgery (m)	Outcome
1	Yamaguchi	2009	53	M	Body	20	DP	21.4	−	12	MPD	None	TP	18	Alive
2	Urata	2012	78	F	Body	22	DP	32	+	34	Parenchyma	None	TP	43	Alive
3	Saeki	2018	54	M	Head	20	PD	20	−	192	MPD/parenchyma	5/5	TP	201	Alive
4	Umemura	2018	53	F	Body	15	DP	None	−	36	MPD	20	TP	84	Alive
5	Our case	2018	60	M	Head	10	PD	35.3	−	16	MPD/floating in MPD (multiple)	20	TP	39	Alive

*TP* total pancreatectomy, *DP* distal pancreatectomy, *PD* pancreatoduodenectomy, *MPD* main pancreatic duct

The primary tumor size may be related to the site of recurrence. In the cases of recurrence in the remnant pancreas, the primary tumors were less than 20 mm, whereas the primary tumors were greater than 90 mm in the cases of recurrence in other regions. The characteristics of these five cases indicated that we should focus on the possibility of recurrence in the remnant pancreas during surveillance after surgery even if the primary ITPN was small and less invasive.

The results of immunohistochemical staining were obtained except case 1 (Table 2). The remaining four cases showed a trend of positive staining for CK7 and CK19 and negative staining for MUC2 and MUC5AC as Yamaguchi et al. [2] described as one of the characteristics of ITPN. The expression of MUC6, which was not included in characteristics of ITPN, was positive in three cases. IPMN is classified into four subtypes, including gastric, intestinal, pancreatobiliary, and oncocytic, based on histomorphological features and immunohistochemical features of mucin glycoproteins [13]. The expression of MUC 6 was positive in three types of IPMN subtypes, including gastric type, pancreatobiliary type, and oncocytic type. Furukawa et al. [14] revealed that the differences in subtypes of IPMN were independent predictors of patient prognosis. Basturk et al. [15] described that the expression of MUC6 supports the presence of a pyloropancreatic pathway distinct from the intestinal pathway in IPMN. Even in ITPN, the biological differences of subtypes, such as IPMN, may be related to the mechanism of implantation into the pancreatic duct.

**Table 2:** Immunohistochemical staining of primary ITPN in five cases that recurred in the remnant pancreas

Case	Author	CK7	CK19	MUC2	MUC6	MUC5AC
1	Yamaguchi	None	None	None	None	None
2	Urata	+	+	+	+	+
3	Saeki	+	+	−	+	−
4	Umemura	+	None	−	+/−	−
5	Our case	+	+	−	−	+/−

Among these five reports of recurrence in the remnant pancreas, four cases of recurrence were found in the remnant main pancreatic duct, and the remaining case was found in the parenchyma of the pancreas. In addition to the presence of residual tumor after surgery, metastasis through the vessel or implantation into the pancreatic duct may cause the recurrence in the remnant pancreas. Each recurrence was observed in the pancreatic duct, indicating a strong possibility of implantation. In our case, no apparent malignancy was confirmed on the pancreatic duct epithelium, while tumor cells floated in the pancreatic duct. These results suggest that the main recurrent lesion would have been caused by tumor cells leaving the tumor and be implanted in the pancreatic duct epithelium.

Regarding ITPN with multicentric occurrence, Kolby et al. [12] reported a case of total pancreatectomy. Kolby et al. initially performed PD for a pancreas head lesion. However, the operative procedure was changed to total pancreatectomy because malignant cells were found in the pancreatic duct during surgery. After surgery, multiple lesions were histopathologically observed in the body and tail of the resected specimen. Although the case reported by Kolby et al. was not a case of recurrence, it seemed reasonable that implantation was the cause of multiple occurrences because tumor cells floated in the pancreatic duct. Our case exhibited recurrence 16 months after the first surgery, which was different from Kolby et al.’s case. Therefore, we should not forget the possibility that the cause of the main recurrent tumor in our case might be a metachronous lesion. However, images and pathological findings did not offer definite evidence whether the recurrent main tumor was due to implantation or a new lesion. Since the growth rate of the ITPN after implantation is unknown, the period from the first surgery to recurrence is not useful to decide the cause of recurrence.

Implantation may occur in any cancer, but cholangiocarcinoma and lung cancer are diseases in which implantations frequently occur. The commonalities between these tumors and ITPN are currently unknown. As a related study, KAJI et al. [16] reported that interleukin induced by pancreatic cancer enhanced the adhesion of cancer cells to epithelial cells. In addition, Date et al. [17] analyzed a mechanism of recurrence of multifocal main duct IPMN after surgery. Date et al. described the development of a secondary IPMN caused by tumor cells obtaining migratory ability and implanted in the ductal epithelium. A similar process also may occur in ITPN and cause implantation into the pancreatic duct.

To summarize these considerations, the cause of ITPN recurrence in our case seems to be due to tumor cells leaving the tumor and implanting into the pancreatic duct.

## Abbreviations

CA19-9: Carbohydrate antigen 19-9
CEA: Carcinoembryonic antigen
CT: Computed tomography
DWI: Diffusion-weighted imaging
ERCP: Endoscopic retrograde cholangiopancreatography
HE: Hematoxylin-eosin staining
IPMN: Intraductal papillary mucinous neoplasm
ITPN: Intraductal tubulopapillary neoplasm
MRI: Magnetic resonance imaging
PD: Pancreaticoduodenectomy
PET-CT: Positron emission tomography/computed tomography
SSPPD: Subtotal stomach-preserving pancreatoduodenectomy
